# Supplementary material for: Evaluating the accuracy of genomic prediction of growth and wood traits in two Eucalyptus species and their F1 hybrids
Source: BMC Plant Biol. 2017 Jun 29;17:110. doi: 10.1186/s12870-017-1059-6 (PMC5492818; doi:10.1186/s12870-017-1059-6)
Supplement: Supplementary file 1 — Average accuracy of SNP imputation methods with increasing proportions of missing data. SNPs on chromosomes 6 and 8 were randomly removed from the dataset to generate specific missing data proportions. Accuracy between imputed and true SNP genotypes were subsequently calculated with the different methods. (DOCX 1714 kb) [file 12870_2017_1059_MOESM1_ESM.docx]

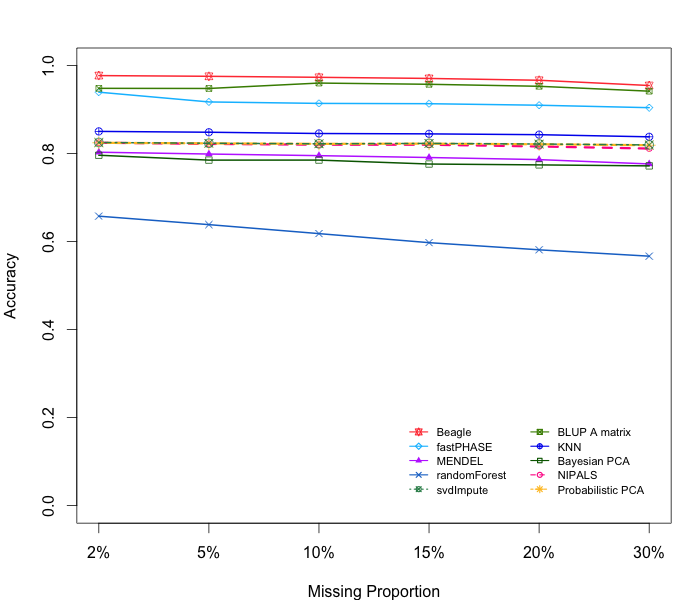


**Additional file 1 Average accuracy of SNP imputation methods with increasing proportions of missing data**. SNPs on chromosomes 6 and 8 were randomly removed from the dataset to generate specific missing data proportions. Accuracy between imputed and true SNP genotypes were subsequently calculated with the different methods.
