# Supplementary material for: Evaluating the accuracy of genomic prediction of growth and wood traits in two Eucalyptus species and their F1 hybrids
Source: BMC Plant Biol. 2017 Jun 29;17:110. doi: 10.1186/s12870-017-1059-6 (PMC5492818; doi:10.1186/s12870-017-1059-6)
Supplement: Supplementary file 3 — ANOVA analysis of sources of variation affecting the predictive ability. (DOCX 48 kb) [file 12870_2017_1059_MOESM3_ESM.docx]

**Additional file 3** ANOVA analysis of sources of variation affecting the predictive ability

| Source^a^ | Degree of Freedom | Sum of Square | Mean Square | F Value | P-value |
| --- | --- | --- | --- | --- | --- |
| TS/VS composition | 3 | 22.3 | 7.44 | 436.68 | < 2e-16 *** |
| TS/VS size | 4 | 21.5 | 5.368 | 315.03 | < 2e-16 *** |
| Method | 3 | 0.3 | 0.085 | 4.97 | 0.00189** |
| Error | 127989 | 2736.9 | 0.017 |  |  |

^a^ Sources of variation were: method (Bayesian LASSO, RKHS, rrBLUP and GBLUP); TS/VS composition (CV_1_, CV_2_, CV_3_ and CV_4_); TS/VS size (558/558, 743/374, 836/281, 892/225 and 1003/114).
