# Supplementary material for: Evaluating the accuracy of genomic prediction of growth and wood traits in two Eucalyptus species and their F1 hybrids
Source: BMC Plant Biol. 2017 Jun 29;17:110. doi: 10.1186/s12870-017-1059-6 (PMC5492818; doi:10.1186/s12870-017-1059-6)
Supplement: Supplementary file 4 — Mean and standard deviation of predictive ability with the five prediction methods for the eight traits. (DOCX 96 kb) [file 12870_2017_1059_MOESM4_ESM.docx]

**Additional file 4** Mean and standard deviation of predictive ability with the five prediction methods for the eight traits. In bold are indicated the highest estimates observed.

|  | ABLUP^4^ | GBLUP | rrBLUP | BL | RKHS |
| --- | --- | --- | --- | --- | --- |
| CBH(3)^1^ | 0.038(0.06)^c2,3^ | 0.117(0.061)^b^ | 0.119(0.068)^b^ | 0.124(0.062)^a^ | **0.124(0.064)**^a^ |
| CBH(6) | 0.145(0.064)^c^ | 0.246(0.066)^b^ | 0.247(0.068)^b^ | 0.246(0.069)^b^ | **0.251(0.066)**^a^ |
| Height(3) | 0.067(0.056)^c^ | 0.179(0.063)^b^ | 0.178(0.063)^b^ | 0.181(0.062)^b^ | **0.198(0.059)**^a^ |
| Height(6) | 0.149(0.067)^c^ | **0.283(0.07)**^a^ | 0.281(0.071)^a^ | 0.282(0.07)^a^ | 0.274(0.064)^b^ |
| Volume(3) | 0.059(0.06)^d^ | 0.149(0.065)^c^ | 0.150(0.064)^c^ | 0.155(0.063)^b^ | **0.176(0.062)**^a^ |
| Volume(6) | 0.113(0.064)^b^ | 0.291(0.063)^a^ | 0.291(0.063)^a^ | 0.292(0.062)^a^ | **0.292(0.062)**^a^ |
| Basic density | 0.168(0.074)^b^ | **0.465(0.055)^a^** | 0.465(0.056)^a^ | 0.464(0.056)^a^ | 0.463(0.058)^a^ |
| Pulp yield | 0.225(0.073)^c^ | **0.433(0.053)^a^** | 0.434(0.053)^a^ | 0.433(0.053)^a^ | 0.406(0.054)^b^ |
| Average | 0.121(0.088)c | 0.27(0.134)b | 0.271(0.134)b | 0.271(0.132)b | 0.274(0.123)a |

^1^ Number in the parentheses represents the age of trait measurement;

^2^ Mean and standard deviation of each method were calculated by taking TS/VS composition and their sizes together;

^3^ Different alphabetic letters indicate significant difference between methods for each trait after one-way ANOVA and further paired t-tests, adjusted by Bonferroni correction;

^4^ Abbreviation: ABLUP, pedigree-base best linear unbiased prediction; GBLUP, genomic BLUP; rrBLUP, random regression BLUP; BL, Bayesian LASSO; RKHS, reproducing kernel Hillbert space.
