# Supplementary material for: Evaluating the accuracy of genomic prediction of growth and wood traits in two Eucalyptus species and their F1 hybrids
Source: BMC Plant Biol. 2017 Jun 29;17:110. doi: 10.1186/s12870-017-1059-6 (PMC5492818; doi:10.1186/s12870-017-1059-6)
Supplement: Supplementary file 5 — Mean and standard deviation of predictive ability estimated with the four Training Set/Validation Set compositions. (DOCX 84 kb) [file 12870_2017_1059_MOESM5_ESM.docx]

**Additional file 5** Mean and standard deviation of predictive ability estimated with the four Training Set/Validation Set compositions

|  | CV_1_^4^ | CV_2_ | CV_3_ | CV_4_ |
| --- | --- | --- | --- | --- |
| CBH(3)^1^ | 0.126(0.056)^b2,3^ | **0.136(0.057)**^a^ | 0.089(0.069)^c^ | 0.127(0.055)^b^ |
| CBH(6) | 0.244(0.065)^b^ | **0.265(0.063)**^a^ | 0.237(0.074)^c^ | 0.244(0.062)^b^ |
| Height(3) | 0.185(0.06)^b^ | **0.193(0.059)**^a^ | 0.17(0.069)^c^ | 0.183(0.059)^b^ |
| Height(6) | 0.279(0.067)^b^ | **0.297(0.064)**^a^ | 0.27(0.076)^c^ | 0.276(0.066)^b^ |
| Volume(3) | 0.161(0.059)^b^ | **0.173(0.06)**^a^ | 0.128(0.073)^c^ | 0.16(0.056)^b^ |
| Volume(6) | 0.287(0.059)^b^ | **0.308(0.057)**^a^ | 0.286(0.073)^b^ | 0.286(0.058)^b^ |
| Basic density | 0.467(0.048)^b^ | **0.487(0.046)**^a^ | 0.435(0.068)^c^ | 0.468(0.045)^b^ |
| Pulp yield | **0.436(0.052)**^a^ | 0.431(0.053)^b^ | 0.41(0.058)^c^ | 0.435(0.05)^a^ |
| **Average** | 0.273(0.13)^b^ | **0.286(0.129)**^a^ | 0.253(0.136)^c^ | 0.272(0.129)^b^ |

^1^ Number in the parentheses represents the age of trait measurement;

^2^ Mean and standard deviation of each TS/VS composition were calculated by taking methods (without ABLUP) and TS/VS sizes together;

^3^ Different alphabetic letters indicate significant difference between TS/VS composition for each trait after one-way ANOVA and further paired t-tests, adjusted by Bonferroni correction;

^4^ Abbreviation: CV_1_, random assignment; CV_2_ all parents are in the TS; CV_3_, distant relatedness between TS and VS; CV_4_, close relatedness between TS and VS.
