# Supplementary material for: Evaluating the accuracy of genomic prediction of growth and wood traits in two Eucalyptus species and their F1 hybrids
Source: BMC Plant Biol. 2017 Jun 29;17:110. doi: 10.1186/s12870-017-1059-6 (PMC5492818; doi:10.1186/s12870-017-1059-6)
Supplement: Supplementary file 6 — Mean and standard deviation of predictive ability estimated with the five relative sizes of Training Set/Validation Set expressed in proportions and numbers of individuals. (DOCX 89 kb) [file 12870_2017_1059_MOESM6_ESM.docx]

**Additional file 6** Mean and standard deviation of predictive ability estimated with the five relative sizes of Training Set/Validation Set expressed in proportions and numbers of individuals

|  | 1:1 (558/558) | 2:1 (743/374) | 3:1 (836/281) | 4:1 (892/225) | 9:1 (1003/114) |
| --- | --- | --- | --- | --- | --- |
| CBH(3)^1^ | 0.102(0.035)^d2,3^ | 0.116(0.046)^c^ | 0.122(0.055)^b^ | 0.127(0.062)^a^ | **0.13(0.093)**^a^ |
| CBH(6) | 0.231(0.035)^d^ | 0.243(0.048)^c^ | 0.251(0.058)^b^ | 0.252(0.069)^b^ | **0.26(0.101)**^a^ |
| Height(3) | 0.16(0.034)^d^ | 0.178(0.043)^c^ | 0.187(0.053)^b^ | 0.19(0.062)^b^ | **0.198(0.096)**^a^ |
| Height(6) | 0.263(0.038)^d^ | 0.276(0.05)^c^ | 0.283(0.061)^b^ | 0.288(0.069)^ab^ | **0.292(0.105)**^a^ |
| Volume(3) | 0.128(0.04)^e^ | 0.15(0.047)^d^ | 0.159(0.056)^c^ | 0.165(0.065)^b^ | **0.176(0.093)**^a^ |
| Volume(6) | 0.277(0.033)^d^ | 0.289(0.045)^c^ | 0.294(0.056)^b^ | 0.296(0.064)^b^ | **0.304(0.095)**^a^ |
| Basic density | 0.445(0.034)^d^ | 0.462(0.043)^c^ | 0.47(0.049)^b^ | 0.471(0.056)^ab^ | **0.475(0.081)**^a^ |
| Pulp yield | 0.403(0.031)^e^ | 0.425(0.039)^d^ | 0.431(0.048)^c^ | 0.437(0.053)^b^ | **0.445(0.079)**^a^ |
| **Average** | 0.251(0.121)^e^ | 0.267(0.125)^d^ | 0.274(0.128)^c^ | 0.279(0.132)^b^ | **0.285(0.148)**^a^ |

^1^Number in the parentheses represents the age of trait measurement;

^2^Mean and standard deviation of predictive ability on each TS/VS relative size are calculated by taking statistical methods (without ABLUP) and TS/VS compositions together;

^3^Alphabetic letters display significant difference between TS/VS size of each trait after one-way ANOVA and further paired t-tests, adjusted by Bonferroni correction.
