# Supplementary material for: Evaluating the accuracy of genomic prediction of growth and wood traits in two Eucalyptus species and their F1 hybrids
Source: BMC Plant Biol. 2017 Jun 29;17:110. doi: 10.1186/s12870-017-1059-6 (PMC5492818; doi:10.1186/s12870-017-1059-6)
Supplement: Supplementary file 9 — ANOVA of predictive ability with SNP genomic location and SNP number as sources of variation. (DOCX 62 kb) [file 12870_2017_1059_MOESM9_ESM.docx]

**Additional file 9** ANOVA of predictive ability with SNP genomic location and SNP number as sources of variation

| Model | Source^1^ | Degree of Freedom | Sum of Square | Mean Square | F Value | P-value |
| --- | --- | --- | --- | --- | --- | --- |
| 1 | **SNP location** | 3 | 2.4 | 0.801 | 47.36 | < 2e-16 *** |
|  | TS/VS composition | 3 | 14.1 | 4.687 | 277.14 | < 2e-16 *** |
|  | Method | 1 | 0.2 | 0.173 | 10.23 | 0.00138** |
|  | Error | 51192 | 342.5 | 0.017 |  |  |
| 2 | **SNP size** | 11 | 803.1 | 73.01 | 4864.8 | <2e-16 *** |
|  | TS/VS composition | 3 | 38.5 | 12.83 | 855.0 | <2e-16 *** |
|  | Method | 1 | 4.32 | 4.3 | 287.7 | <2e-16 *** |
|  | Error | 153584 | 4034 | 0.02 |  |  |

^1^ Sources of variation are: method (RKHS and GBLUP); TS/VS composition (CV_1_, CV_2_, CV_3_ and CV_4_); SNP location (coding region, gene region, intergenic region, all); SNP number (10, 20, 50, 100, 200, 500, 1000, 2000, 5000, 10000, 20000 and 41304).
