# Supplementary material for: Evaluating the accuracy of genomic prediction of growth and wood traits in two Eucalyptus species and their F1 hybrids
Source: BMC Plant Biol. 2017 Jun 29;17:110. doi: 10.1186/s12870-017-1059-6 (PMC5492818; doi:10.1186/s12870-017-1059-6)
Supplement: Supplementary file 10 — Average predictive ability estimated with different numbers of SNPs fitted into the model. (DOCX 136 kb) [file 12870_2017_1059_MOESM10_ESM.docx]

**Additional file 10** Average predictive ability estimated with different numbers of SNPs fitted into the model

| Trait | 40K^4^ | 20K | 10K | 5K | 2K | 1K | 500 | 200 | 100 | 50 | 20 | 10 |
| --- | --- | --- | --- | --- | --- | --- | --- | --- | --- | --- | --- | --- |
| GBLUP | | | | | | | | | | | | |
| CBH (3)^1^ | 0.127^a2,3^ | 0.124^a^ | 0.121^a^ | 0.121^a^ | 0.112^b^ | 0.104^c^ | 0.096^d^ | 0.08^e^ | 0.068^f^ | 0.052^g^ | 0.036^h^ | 0.024^i^ |
| CBH (6) | 0.251^a^ | 0.253^a^ | 0.249^a^ | 0.249^a^ | 0.238^b^ | 0.229^c^ | 0.214^d^ | 0.188^e^ | 0.167^f^ | 0.145^g^ | 0.111^h^ | 0.086^i^ |
| Height (3) | 0.186^a^ | 0.182^ab^ | 0.179^ab^ | 0.177^bc^ | 0.171^c^ | 0.157^d^ | 0.146^e^ | 0.128^f^ | 0.115^g^ | 0.103^h^ | 0.084^i^ | 0.066^j^ |
| Height (6) | 0.291^a^ | 0.291^a^ | 0.288^a^ | 0.287^a^ | 0.276^b^ | 0.269^b^ | 0.254^c^ | 0.232^d^ | 0.217^e^ | 0.193^g^ | 0.16^h^ | 0.128^i^ |
| Volume (3) | 0.159^a^ | 0.158^a^ | 0.152^b^ | 0.154^ab^ | 0.143^c^ | 0.132^d^ | 0.118^e^ | 0.099^f^ | 0.084^g^ | 0.072^h^ | 0.053^i^ | 0.038^j^ |
| Volume (6) | 0.296^a^ | 0.297^a^ | 0.296^a^ | 0.291^ab^ | 0.284^b^ | 0.276^c^ | 0.265^d^ | 0.244^e^ | 0.221^f^ | 0.202^g^ | 0.167^h^ | 0.137^i^ |
| Basic density | 0.472^a^ | 0.47^a^ | 0.467^a^ | 0.46^b^ | 0.449^c^ | 0.436^d^ | 0.417^e^ | 0.389^f^ | 0.37^g^ | 0.343^h^ | 0.302^i^ | 0.252^j^ |
| Pulp yield | 0.443^a^ | 0.441^a^ | 0.437^b^ | 0.428^c^ | 0.415^d^ | 0.394^e^ | 0.371^f^ | 0.326^g^ | 0.29^h^ | 0.256^i^ | 0.207^j^ | 0.169^k^ |
| RKHS | | | | | | | | | | | | |
| CBH (3) | 0.129^a^ | 0.129^a^ | 0.13^a^ | 0.126^ab^ | 0.127^ab^ | 0.117^b^ | 0.112^c^ | 0.098^d^ | 0.088^e^ | 0.075^f^ | 0.054^g^ | 0.036^h^ |
| CBH (6) | 0.257^ab^ | 0.261^a^ | 0.256^ab^ | 0.257^ab^ | 0.245^bc^ | 0.24^c^ | 0.23^d^ | 0.209^e^ | 0.186^f^ | 0.161^g^ | 0.12^h^ | 0.088^i^ |
| Height (3) | 0.203^a^ | 0.203^a^ | 0.205^a^ | 0.204^a^ | 0.196^ab^ | 0.186^b^ | 0.171^c^ | 0.155^d^ | 0.138^e^ | 0.12^f^ | 0.092^g^ | 0.071^h^ |
| Height (6) | 0.281^ab^ | 0.284^a^ | 0.284^a^ | 0.276^ab^ | 0.271^bc^ | 0.264^c^ | 0.259^d^ | 0.239^e^ | 0.221^f^ | 0.197^g^ | 0.15^h^ | 0.119^i^ |
| Volume (3) | 0.183^a^ | 0.181^a^ | 0.178^ab^ | 0.179^ab^ | 0.168^bc^ | 0.164^c^ | 0.155^d^ | 0.133^e^ | 0.125^f^ | 0.1^g^ | 0.076^h^ | 0.059^i^ |
| Volume (6) | 0.301^a^ | 0.299^ab^ | 0.298^ab^ | 0.294^abc^ | 0.288^bc^ | 0.286^c^ | 0.271^d^ | 0.254^e^ | 0.229^f^ | 0.208^g^ | 0.168^h^ | 0.129^i^ |
| Basic density | 0.468^a^ | 0.471^a^ | 0.468^a^ | 0.464^ab^ | 0.455^bc^ | 0.449^c^ | 0.436^d^ | 0.409^e^ | 0.384^f^ | 0.352^g^ | 0.298^h^ | 0.239^i^ |
| Pulp yield | 0.414^a^ | 0.413^a^ | 0.407^ab^ | 0.409^ab^ | 0.4^b^ | 0.386^c^ | 0.369^d^ | 0.339^e^ | 0.307^f^ | 0.269^g^ | 0.205^h^ | 0.159^i^ |
| **Average** | **0.278^a^** | **0.278^a^** | **0.275^ab^** | **0.272^b^** | **0.263^c^** | **0.253^d^** | **0.239^e^** | **0.216^f^** | **0.197^g^** | **0.175^h^** | **0.142^i^** | **0.113^j^** |

^1^ Number in the parentheses represents the age of the traits measurement;

^2^ The average of predictive ability on each number of SNPs across two methods is calculated by comprising four TS/VS compositions;

^3^ Alphabetic letters display significant difference between SNP sizes of each trait and method after one-way ANOVA and further paired t-tests, adjusted by Bonferroni correction;

^4^ SNP numbers: 40K = 40000 SNPs and else.
