# Supplementary material for: Evaluating the accuracy of genomic prediction of growth and wood traits in two Eucalyptus species and their F1 hybrids
Source: BMC Plant Biol. 2017 Jun 29;17:110. doi: 10.1186/s12870-017-1059-6 (PMC5492818; doi:10.1186/s12870-017-1059-6)
Supplement: Supplementary file 11 — Average predictive abilities estimated using SNP sets located in different genomic regions. (DOCX 82 kb) [file 12870_2017_1059_MOESM11_ESM.docx]

**Additional file 11** Average predictive abilities estimated using SNP sets located in different genomic regions

|  | **All**  **(41,304 SNPs)** | **Coding region (11,786 SNPs)** | **Genic region (30,405 SNPs)** | **Intergenic**  **(10,899 SNPs)** |
| --- | --- | --- | --- | --- |
| **GBLUP** |  |  |  |  |
| CBH(3)^1^ | 0.127^b2,3^ | 0.117^c^ | 0.119^c^ | 0.138^a^ |
| CBH(6) | 0.251^NS^ | 0.248^NS^ | 0.247^NS^ | 0.25^NS^ |
| Height(3) | 0.186^b^ | 0.165^d^ | 0.176^c^ | 0.206^a^ |
| Height(6) | 0.29^ab^ | 0.282^b^ | 0.286^b^ | **0.298**^a^ |
| Volume(3) | 0.159^b^ | 0.149^c^ | 0.151^c^ | 0.169^a^ |
| Volume(6) | 0.296^NS^ | 0.295^NS^ | 0.296^NS^ | 0.293^NS^ |
| Basic density | 0.472^ab^ | 0.466^b^ | 0.467^b^ | **0.474**^a^ |
| Pulp yield | **0.443**^a^ | 0.436^ab^ | 0.441^a^ | 0.432^b^ |
| **RKHS** |  |  |  |  |
| CBH (3) | 0.129^b^ | 0.126^b^ | 0.127^b^ | **0.143**^a^ |
| CBH (6) | 0.257^ab^ | 0.248^b^ | 0.256^ab^ | **0.267**^a^ |
| Height (3) | 0.203^b^ | 0.186^c^ | 0.197^bc^ | **0.226**^a^ |
| Height (6) | 0.281^b^ | 0.273^c^ | 0.279^b^ | 0.292^a^ |
| Volume (3) | 0.186^a^ | 0.168^c^ | 0.173^b^ | **0.192**^a^ |
| Volume (6) | 0.301^ab^ | 0.296^b^ | 0.295^b^ | **0.307**^a^ |
| Basic density | 0.468^NS^ | 0.467^NS^ | 0.467^NS^ | 0.47^NS^ |
| Pulp yield | 0.414^NS^ | 0.41^NS^ | 0.411^NS^ | 0.408^NS^ |
| **Average** | 0.279^b^ | 0.270^d^ | 0.274^c^ | 0.284^a^ |

^1^ Number in the parentheses represents the age of trait measurement;

^2^ Average predictive ability for each SNP location across the two method and four TS/VS genetic compositions;

^3^ Letters indicate significant difference between SNP locations after one-way ANOVA and further paired t-tests, adjusted by Bonferroni correction. NS indicate non-significant difference.
