# Supplementary material for: Evaluating the accuracy of genomic prediction of growth and wood traits in two Eucalyptus species and their F1 hybrids
Source: BMC Plant Biol. 2017 Jun 29;17:110. doi: 10.1186/s12870-017-1059-6 (PMC5492818; doi:10.1186/s12870-017-1059-6)
Supplement: Supplementary file 12 — Decay of linkage disequilibrium (LD) with physical distance estimated with SNPs in different genomic locations. (a) A comparison of the decay of LD with physical distance in four classes of SNPs located with coding, genic, intergenic and all regions, respectively. Dots of pairwise LD versus physical distance and the LD decay for SNPs located in all regions (b), coding region (c), genic region (d) and intergenic region (e), respectively. (DOCX 1375 kb) [file 12870_2017_1059_MOESM12_ESM.docx]

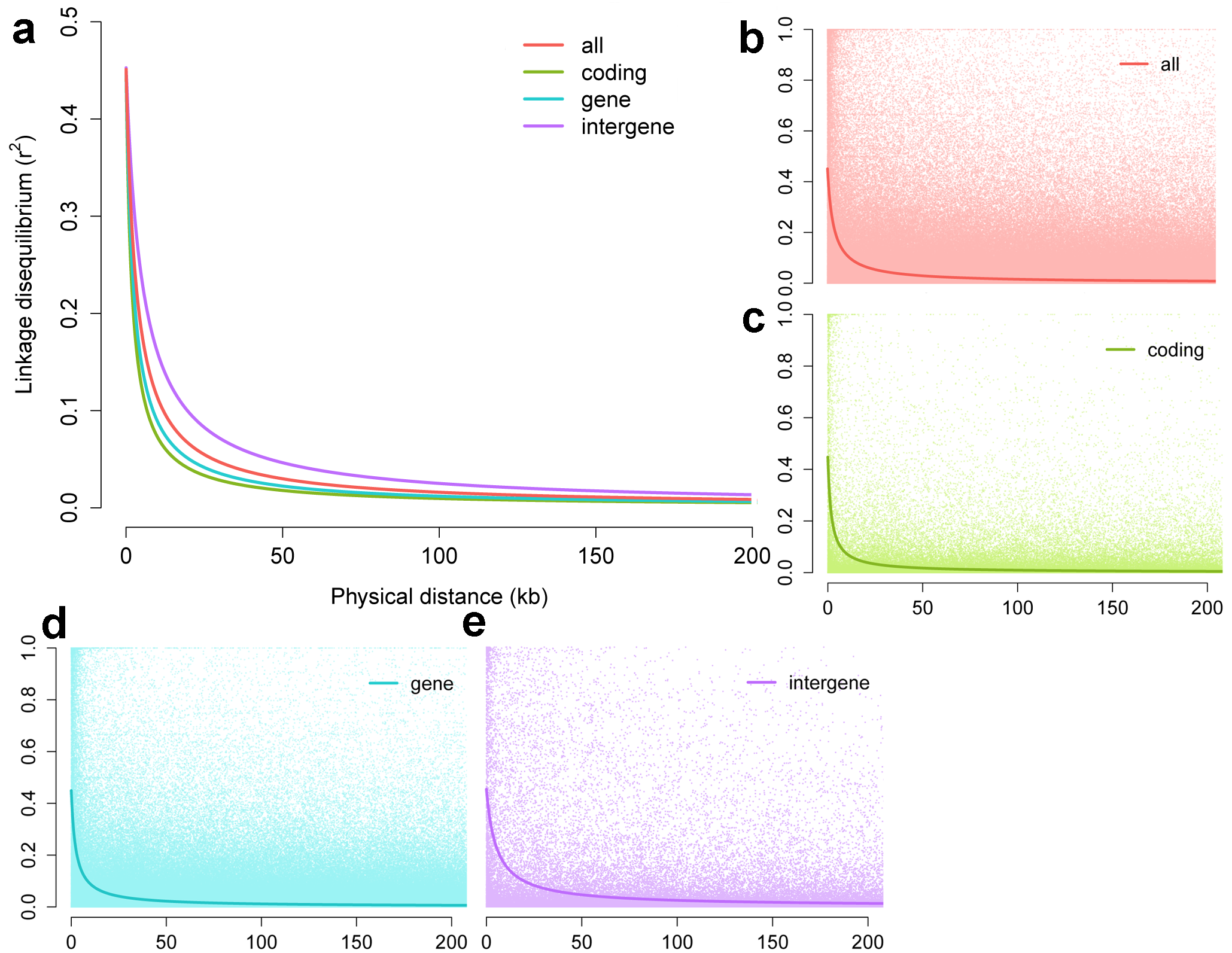


**Additional file 12** **Decay of linkage disequilibrium (LD) with physical distance estimated with SNPs in different genomic locations.** (a) A comparison of the decay of LD with physical distance in four classes of SNPs located with coding, genic, intergenic and all regions, respectively. Dots of pairwise LD versus physical distance and the LD decay for SNPs located in all regions (b), coding region (c), genic region (d) and intergenic region (e), respectively.
