# Supplementary material for: Evaluating the accuracy of genomic prediction of growth and wood traits in two Eucalyptus species and their F1 hybrids
Source: BMC Plant Biol. 2017 Jun 29;17:110. doi: 10.1186/s12870-017-1059-6 (PMC5492818; doi:10.1186/s12870-017-1059-6)
Supplement: Supplementary file 13 — Predictive abilities by training in pure species eucalypt parents and predicting in their F1 hybrids. Predictive ability estimated under three training/validation sets (TS/VS) scenarios with two methods (GBLUP and RKHS) for each trait. PO168 (red boxes): all 168 E. grandis and E. urophylla pure species G0 parents used for training and 168 G1 random selected hybrid progeny for validation; random168 (green): randomly selected 168 individuals from all 1117 for TS and 168 randomly also for VS; random558 (blue): randomly divided all 1117 individuals into TS and VS of same size (558/558). Outlier estimates are indicated by black dots. (DOCX 174 kb) [file 12870_2017_1059_MOESM13_ESM.docx]

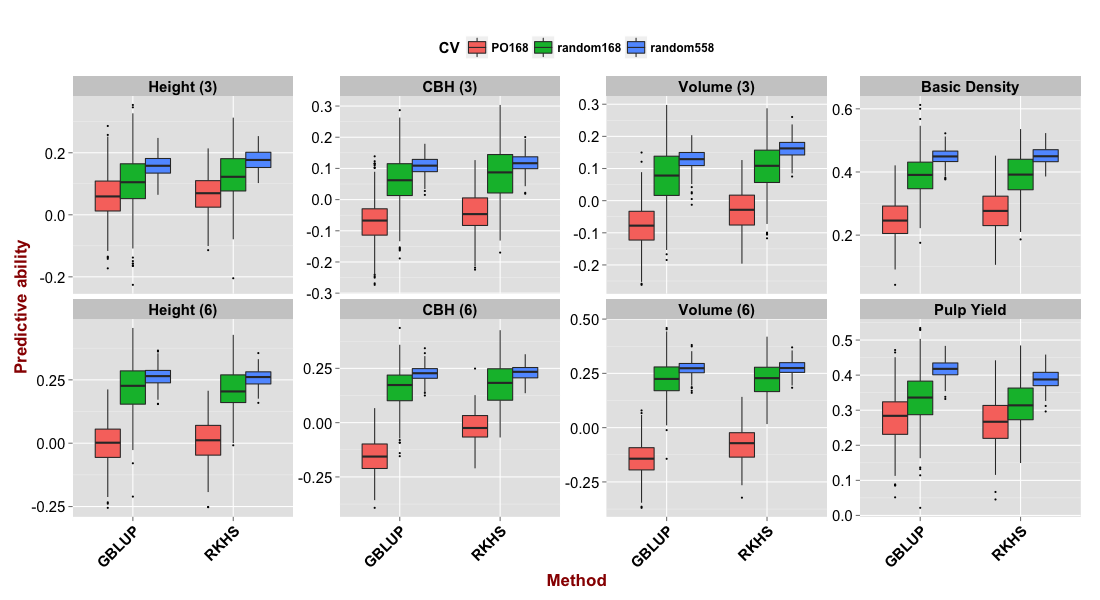


**Additional file 13** **Predictive abilities by training in pure species eucalypt parents and predicting in their F_1_ hybrids**. Predictive ability estimated under three training/validation sets (TS/VS) scenarios with two methods (GBLUP and RKHS) for each trait. PO168 (red boxes): all 168 *E. grandis* and *E. urophylla* pure species G0 parents used for training and 168 G1 random selected hybrid progeny for validation; random168 (green): randomly selected 168 individuals from all 1,117 for TS and 168 randomly also for VS; random558 (blue): randomly divided all 1,117 individuals into TS and VS of same size (558/558). Outlier estimates are indicated by black dots.
